# The perfect storm in a coastal pond: duckweed, botulism, bird mortalities, and recurrent blooms of *Euglena sanguinea*

**DOI:** 10.1007/s11356-026-37791-z

**Published:** 2026-05-08

**Authors:** Francisco Rodríguez, Begoña Ben-Gigirey, Pablo Caballero, Jose Luis Garrido, Lucía Viñas, Pilar Riobó, María García-Portela

**Affiliations:** 1 Centro Nacional Instituto Español de Oceanografía (IEO-CSIC), Centro Oceanográfico de Vigo, 36390 Vigo, Spain; 2 Servicio de Patrimonio Natural de Pontevedra (Consellería de Medio Ambiente E Cambio Climático-Xunta de Galicia), 36003 Pontevedra, Spain; 3 Instituto de Investigacións Mariñas, Consejo Superior de Investigaciones Científicas (IIM-CSIC), 36208 Vigo, Spain

**Keywords:** Eutrophication, Avian botulism, *Anas platyrhynchos*, *Lemna minor*, *Euglena sanguinea*, Euglenophycin, Organic pollutants, A Congorza

## Abstract

In summer 2024, a freshwater coastal pond in northwest Spain (A Congorza, Cangas) experienced its first massive mallard (*Anas platyrhynchos*) mortalities, repeated in 2025. The pond is covered by *Lemna minor* and develops a red scum during summer. Molecular, pigment, and morphological analyses allowed identification of *Euglena sanguinea* as the responsible species for the latter. Water, tissues, and maggots from dead specimens were analyzed to assess physico-chemical characteristics, plankton composition, clinical signs, and toxins in order to explain these mortalities. Poor water quality parameters were determined during summer 2025 with suboxic dissolved oxygen (1.5–5.2 mg L⁻^1^) and high levels of biological and chemical oxygen demand (17–130 and 56–535 mg L⁻^1^, respectively) revealed severe degradation, failing to meet Directive EU/2024/3019 standards. Legacy persistent organic pollutants (PAHs, PCBs, OCPs, PBDEs) appeared at low, non-lethal levels. Clinical signs and analyses confirmed avian botulism (type C and C/D toxins) as the primary cause of death. LC-HRMS detected putative euglenophycin isomers in *E. sanguinea* pellets. Overall, these findings indicate that avian botulism is the primary cause of mallard mortalities in A Congorza. The dominance of *L. minor* and recurrent *E. sanguinea* red blooms evidence the eutrophication and severe habitat degradation potentially contributing to a multifactorial stress fatal to *A. platyrhynchos*.

**Supplementary Information:**

The online version contains supplementary material available at 10.1007/s11356-026-37791-z.

## Introduction

Permanent wetlands such as lakes and some ponds play a crucial role for ecosystem services and biodiversity providing food and shelter for wildlife like amphibians, reptiles, fish, and waterfowl (Gopal 2009; Maltby and Barker 2009). However, these environments are highly susceptible to nutrient enrichment, often resulting in eutrophication that can alter community composition, reduce biodiversity, and impair ecosystem functioning (Sánchez-Carrillo et al. 2010). Furthermore, eutrophication can result in other undesirable changes, such as the onset of anaerobic conditions, favouring the growth of *Clostridium botulinum* and botulism outbreaks of special concern for waterbirds (Anza et al. 2016; Mena-Casero et al. 2025; Vidal et al. 2013).

Biological indicators of nutrient enrichment in wetlands include shifts in plant composition and growth (e.g. overgrowth of aquatic plants such as *Lemna*, known as duckweed), as well as in the microbial communities (phytoplankton blooms often associated with toxic cyanobacteria; Akinnawo 2023; Sims et al. 2013). Protists such as diatoms, chlorophytes, and euglenophytes have also been applied in biotic indices to assess water quality and organic contamination (Guo et al. 2010; Sims et al. 2013; Venkatachalapathy and Karthikeyan 2015).

Among these indicators, euglenophytes (Protozoa, Discoba), particularly *Euglena sanguinea*, have gained attention as indicators of organic pollution and water quality deterioration (Janse van Vuuren and Levanets 2021; Sawaiker and Rodrigues 2023; Janse van Vuuren et al 2025). These mixotrophic organisms are widely distributed in freshwater, estuarine, and sedimentary habitats and can form conspicuous reddish blooms due to the accumulation of secondary carotenoids such as astaxanthin (Laza-Martínez et al. 2019). Importantly, *E. sanguinea* can produce euglenophycin, a toxin with ichthyotoxic, herbicidal, and algaecidal effects, which has been linked to fish mortalities in aquaculture and may pose risks to natural food webs (Cabang et al. 2017; Zimba et al. 2004).

A Congorza is a freshwater coastal pond in NW Spain that exhibits recurrent reddish scums since summer 2021 together with dense mats of *Lemna minor*. By summer 2024, mortalities of mallards (*Anas platyrhynchos*) were first observed, prompting investigations by regional authorities and our group at IEO-CSIC, in collaboration with the Wildlife Rescue Center (WRC) of Carballedo (Xunta de Galicia), to determine the origin of these deaths and potential sanitary risks. Moreover, a paralytic syndrome was observed in rescued mallards associated with the presence of a reddish scum on the surface of the pond.

To investigate the possible causes of the mortalities and clinical signs in mallards, plankton, and freshwater samples, as well as tissues from affected birds and maggots collected from carcasses, were analyzed using a combination of microscopic and chemical approaches, including liquid chromatography high-resolution mass spectrometry (LC-HRMS). The aim of this study was to determine the potential biological and toxicological factors involved in the observed mortality event, and to provide information that may assist in the management and protection of the aquatic ecosystem and wildlife of the studied pond and other similar environments.

## Materials and methods

### Description of sampling location

**Fig. 1:**
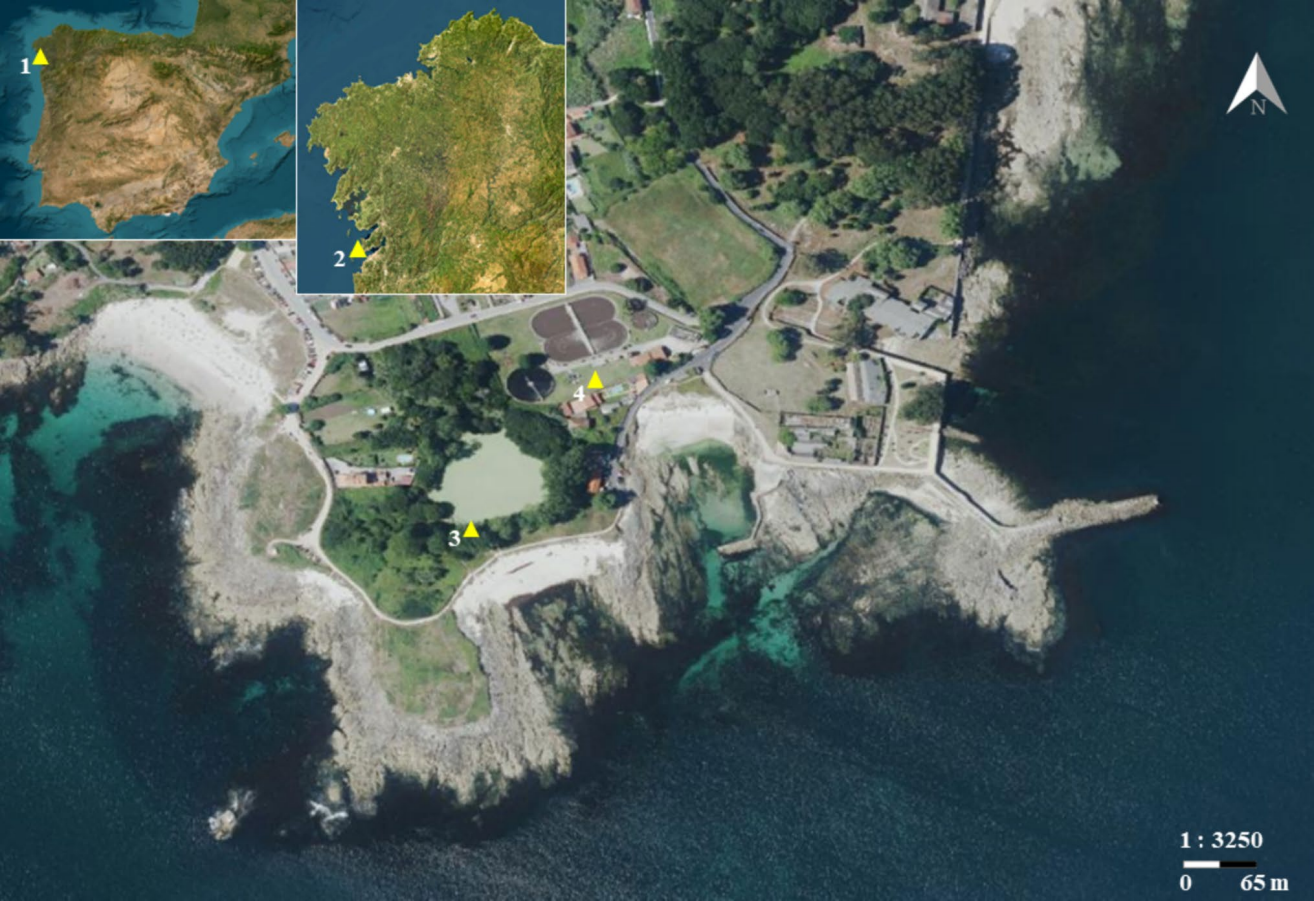
Location of A Congorza Lagoon in NW Spain. Yellow triangles indicate the position of the lagoon at different spatial scales: (1) within the Iberian Peninsula, (2) within Galicia, and (3) its exact location in Punta Balea, Darbo (Cangas do Morrazo). Triangle (4) marks the Cangas Wastewater Treatment Plant (EDAR), situated near A Congorza Lagoon at Muelle da Congorza

The pond of A Congorza is located in Cangas do Morrazo (42°14′56″N, 8°47′33″W), in the northern coastline of Ría de Vigo (Fig. 1). It represents one of the few permanent freshwater reservoirs in the province of Pontevedra, surrounded by diverse vegetation (marsh plants and a small forest of willows and laurels), beaches, and a coastal trail that receives many visitors throughout the year, especially in summer. Nearby, there is a water treatment plant (Fig. 1, triangle 4) and the ruins of an old canning factory (Massó Hermanos S.A.). A Congorza is a natural wetland deepened by the Massó family in the early XX century. They turned it into a freshwater coastal pond, with a surface of 2.6 ha with a sheet of water of 6000 m^2^ and a flat bottom with maximum depth of 2–3 m. Among its fauna stands out the presence of waterfowl, like the common moorhen (*Gallinula chloropus*), Eurasian coot (*Fulica atra*), mallards (*A. platyrhynchos*), kingfishers (*Alcedo atthis*), and little egret and gray herons (*Egretta garzetta* and *Ardea cinerea*) (Fernández Cordeiro et al. 2006).

Nowadays, the surface of the pond is mostly covered by a thick mat of the common duckweed *L. minor* and a reddish scum of *E. sanguinea*, only sporadically dispersed by storms or strong northerlies in spring and summer.

### Environmental conditions and sampling

Air temperature at 1.5 m height and pluviosity were obtained from MeteoGalicia weather and forecast services (meteogalicia.gal/web/observacion/rede-meteoroloxica) for the period 2016–2025 for the meteorological station located at the port of Cangas (42°15′38″N, 8°46′57″W). Physico-chemical parameters from surface waters in A Congorza (pH, water temperature, and nutrients) were obtained by Xunta de Galicia (Rede de Observación Ambiental de Galicia (ROAGA), Laboratorio de Medio Ambiente de Galicia (LMAG) and Servizo de Patrimonio Natural de Pontevedra). In addition, water samples for nutrients were directly collected by the IEO-CSIC research team in July 2025 on the shore surface. In August 2025, additional samples were collected by rowing a zodiac (Servizo de Patrimonio Natural de Pontevedra) to the centre of the pond, where a Niskin bottle was used to reach the bottom (2 m depth). These nutrient samples were stored in an ice-filled container and transported immediately to the laboratory.

Surface water samples for biological analyses were collected on the shore of A Congorza in summer 2021, 2024, and 2025 using plastic flasks, harvesting both the green and red scum of *E. sanguinea* stuck to *L. minor* leaves. In order to separate the planktonic organisms, samples were prefiltered through 500 µm mesh to remove leaves, larvae, and gross debris. Subsequently, the filtered material was distributed into suitable containers for pigment and toxin analyses, refrigerated and placed into insulated bags. *Lemna minor* leaves were cleaned up further with distilled water through the same mesh and kept at −20 °C until analysis.

### Nutrient analyses

Surface water samples for nutrients were analyzed between May 29 and Sep 1 2025 by ROAGA-LMAG (Xunta de Galicia), which also assessed a range of water quality parameters (Table 1). In addition, surface and 2 m depth water samples (as formerly detailed) were analyzed by Instituto de Investigacións Mariñas (IIM-CSIC) and Instituto Tecnolóxico para o Control do Medio Mariño en Galicia (INTECMAR, Xunta de Galicia) in 2025. In these cases, prefiltered water samples were passed through Whatman GF/F glass fibre filters and immediately frozen until further analysis. The analytical procedures followed those of Doval et al. (2016) for samples processed by INTECMAR, the corresponding internal laboratory SOP (1063) for ROAGA-LMAG, and the methods of Hansen and Grasshoff (1983) for inorganic nutrients (with ammonium determined according to Kérouel and Aminot 1997) at IIM-CSIC.

**Table 1:** Water quality parameters in surface waters of the pond of A Congorza. Source: ROAGA-LMAG (Xunta de Galicia)

Date	BOD (mg L^−1^)	COD (mg L^−1^)	DO (mg L^−1^)	TSS (mg L^−1^)	pH	NH_4_^+^ (mg L^−1^)	NO_2_^−^ (mg L^−1^)	NO_3_^−^ (mg L^−1^)	TN (mg L^−1^)	TP (mg L^−1^)
29/05/2025	17	56	1.58	36	6.7	< 2.0	< 0.03	< 1.1	2.1	0.2
04/06/2025	25	78	3.91	43	6.7	< 0.5	< 0.03	< 1.1	2.9	0.2
05/06/2025	19	63	2.87	32	6.5	< 0.5	< 0.03	< 1.1	2.7	0.2
11/06/2025	21	71	3.83	38	6.5	< 0.5	< 0.03	< 1.1	2.4	0.2
24/06/2025	20	117	4.72	30	6.8	< 0.5	< 0.03	< 1.1	3.0	0.3
30/06/2025	26	118	5.2	34	6.6	< 0.5	< 0.03	< 1.1	6.0	0.4
09/07/2025	130	535	0.87	320	6.7	< 0.5	< 0.03	< 1.1	18.0	1.7
16/07/2025	25	67	4.9	28	6.7	< 0.5	< 0.03	< 1.1	1.5	0.2
21/07/2025	22	70	5.2	13	6.9	< 0.5	< 0.03	< 1.1	2.0	0.3
29/07/2025	30	88	3.07	40	6.8	< 0.5	< 0.03	< 1.1	5.0	0.6
05/08/2025	60	338	0.85	160	6.7	0.6	< 0.03	< 1.1	7.0	0.8
12/08/2025	40	97	3.74	96	6.8	< 0.5	< 0.03	< 1.1	8.0	0.7
19/08/2025	90	431	2.95	138	6.9	< 0.5	< 0.03	< 1.1	7.0	0.5
26/08/2025	21	58	4.4	53	6.7	< 0.5	< 0.03	< 1.1	5.0	0.4
01/09/2025	20	63	4.09	32	6.7	< 0.5	< 0.03	< 1.1	3.2	0.3
08/09/2025	24	71	3.09	23	6.8	< 0.5	< 0.03	< 1.1	2.6	0.3

### Plankton analysis

Phytoplankton species composition was examined using 1–2 mL Lugol’s solution-fixed subsamples observed with a Nikon Eclipse Ts2 inverted microscope (Nikon Corporation, Tokyo, Japan) for identification of dominant taxa at the IEO-CSIC and ROAGA-LMAG in samples from summer 2021, 2024, and 2025. The identification of dominant taxa was based on visual estimation of relative abundance. Specimens of *E. sanguinea* were isolated in 2024 and 2025 in multi-well plates but efforts to maintain them in liquid medium following Laza-Martínez et al. (2019) proved unsuccessful. Images were captured with a Nikon 4 K full HD autofocus camera coupled to the microscope. Cell measurements of *E. sanguinea* palmeloid (n = 23) and flagellated (n = 44) stages (length and width) were performed on micrographs of Lugol’s solution–fixed samples using the free software ImageJ (Schneider et al. 2012). Mean values are reported with their corresponding standard deviations.

### Molecular analysis of *Euglena sanguinea*

A total of 12 single cells of *Euglena sanguinea* from two independent samplings in July and August 2025 were isolated using a glass capillary and transferred into a 1-mL Eppendorf tube. Cells were pelleted by centrifugation (2 min, 13,000 × *g*), and 100 µL of 10% Chelex® 100 (Sigma-Aldrich) was added. The suspension was centrifuged again (2 min, 13,000 × *g*) and incubated in a heat block at 99 °C for 10 min. Following incubation, the sample was briefly vortexed, centrifuged, and subjected to a second 10-min boiling step at 99 °C. After a final round of vortexing and centrifugation, the supernatant—containing the extracted DNA—was carefully transferred to a fresh Eppendorf tube.

DNA extracts were then used as templates in a nested PCR targeting a fragment of the nuclear small subunit ribosomal DNA (nSSU rDNA, 18S). Amplification was carried out using *E. sanguinea*-specific primers (Kulczycka et al. 2018), with the external primer pair sangF0/sangR0 and the internal primer pair sangF1/sangR1.

PCR amplifications were performed using Horse-Power™ Taq DNA Polymerase MasterMix 2x (Canvax, Spain) following manufacturer’s instructions. The first PCR conditions were as follows: initial denaturation for 5 min at 94 °C, followed by 35 cycles of 35 s at 94 °C, 35 s at 62 °C (sangF0/sangR0) and 3 min at 72 °C, followed by a final extension for 7 min at 72 °C. The second PCR followed the same conditions as the first one but the annealing temperature was 60 °C using primers sangF1/sangR1 and 1 µL of the first PCR product.

The PCR products were purified with ExoSAP–IT (USB Corp., USA), sequenced using Big Dye Terminator v3.1 (Applied Biosystems, USA) and migrated in a SeqStudio genetic analyzer (both at Applied Biosystems, USA) at the CACTI sequencing facilities (Universidade de Vigo).

### Alignment and phylogenetic analysis

Two sequences obtained for the partial SSUrDNA region of *E. sanguinea* in A Congorza (Acc. Nos. PX286749, PX286750) were initially checked using Mega X (Kumar et al. 2018) before further analysis with Geneious Prime® 2024.0.7. The similarity of the obtained rDNA sequence to other available sequences in GenBank was determined by the NCBI BLAST algorithm (Camacho et al. 2009). The final alignment included 55 *Euglena* sequences obtained from GenBank and 2 from *Lepocinclis* as outgroups (764 bp in length). Multiple sequence alignment was performed using the MAFFT v7.490 (Katoh and Standley 2013) plugin within Geneious Prime® using the default auto settings. Both ends of the aligned sequences were trimmed to produce uniform fragment lengths.

The General Time Reversible (GTR+G) model with a gamma distribution parameter was selected using MEGA X software (Kumar et al. 2018) based on the lowest BIC (Bayesian Information Criterion) values. Genetic identity (*p*-distances) were calculated on Geneious Prime®. Phylogenetic trees were inferred using both Bayesian inference (BPI) and Maximum Likelihood (ML) methods. Bayesian posterior probabilities (PP) were estimated with MrBayes 3.2.6 (Huelsenbeck and Ronquist 2001). The following settings were applied: random starting tree, 1 million generations, burn-in of 100,000 generations, four heated chains with a temperature of 0.2, and subsampling every 200 generations. ML analysis was conducted using PHYML with 1000 bootstrap replicates (BS; Guindon and Gascuel 2003). A PP ≥ 0.90 was considered strong support for a clade, and BS values ≥ 70% were considered significant.

### HPLC pigment analysis

As soon as the samples arrived at the laboratory in Vigo, 10 mL subsamples were filtered under reduced vacuum onto glass-fiber filters (GF/F, Whatman Inc.). The filters were immediately frozen at −80 °C until their analysis by HPLC. Pigment extraction and analysis were carried out following Zapata et al. (2000) protocol. All sample preparation procedures were performed under dim green light. The HPLC system was a Waters 2690 Alliance separations module with a Waters 996 photodiode array detector (350–700 nm; 1.2 nm of optical resolution). The stationary phase was a C8 column (Waters Symmetry, 150 × 4.6 mm, 3.5 µm particle size) thermostated at 25 °C. The mobile phases were: A: methanol, B: acetonitrile, C: aqueous pyridine solution (0.025 M pyridine, pH adjusted to 5.0 with acetic acid), and D: acetone. The quaternary elution gradient consisted of a modification of the one proposed by Zapata et al. (2000), extending the analysis time under non-aqueous conditions, from minute 45 onwards, to ensure the elution of the most nonpolar esters (supplementary Table S1). Flow rate was 1 mL min^−1^. The identification was based on the comparison of the retention time and spectral characteristics of peaks with those from commercial standards, extracts from reference cultures and pigments occurring in the genus *Euglena* described in previous papers.

### Toxin and bird disease analyses

The presence of botulinum neurotoxins (BoNT) and other bird diseases in mallard tissues, as well as cyanotoxins and euglenophycin in water and plankton samples, was checked as follows.

### BoNT analyses

In 2024 and 2025, several specimens of *A. platyrhynchos* were observed in bad condition with typical symptoms of flaccid paralysis or already dead (floating carcasses) in A Congorza (Table 2). Affected birds and dead individuals were collected by Xunta de Galicia and examined at WRC of Carballedo to study their clinical signs, necropsy report, and sample collection for toxins. Regarding the latter, liver, encephalon, and digestive contents from several mallards were pooled, homogenized, and frozen until further toxin analyses. These were performed in two different laboratories: LABOKLIN (Alcobendas, Spain) in 2024 and the Laboratorio Central de Veterinaria de Algete (National Reference Laboratory for several animal diseases in Spain) in 2025, by means of an ELISA assay. In addition, the latter laboratory also tested for other bird diseases (West Nile fever (WNF), Newcastle disease, and avian influenza).

**Table 2:** Details about specimens of *Anas platyrhynchos* collected in A Congorza during 2024 and 2025. Source: Xunta de Galicia

Collection date	Number of specimens	Observations
2024		
August 2	6 (dead)	Positive for BoNT
August 4	1 (alive)	Recovered from illness and released on August 28
October 8	2 (dead)	Positive for BoNT
2025		
May 28	1 (dead)	Positive for BoNT
June 5	1 (dead)	Positive for BoNT
June 23	2 (dead)	
June 26	7 (5 dead and 2 alive)	Those alive died on June 26 (euthanasia) and 27

### Cyanotoxin analyses

Water samples collected in August 2024 were analyzed for cyanotoxins by means of LC-MS/MS following Pekar et al. (2016) by the Laboratorio de Saúde Pública de Galicia (Xunta de Galicia).

### Euglenophycin analyses

Water samples with red scum due to *E. sanguinea* and mallard tissues were analyzed for the potential presence of euglenophycin by LC-HRMS at IEO-CSIC as follows:

#### Chemicals

Unless otherwise stated, chemicals used were LC-MS-reagent grade. Acetonitrile (MeCN) and formic acid were Optima™ grade from Fisher Scientific (France); LiChrosolv® water (Supelco) was purchased from Merck (Spain). HiperSolv Chromanorm Methanol (MeOH) was from VWR Chemicals (Spain).

No euglenophycin standard is commercially available.

#### Water samples processing

In August 2025, a surface water sample previously filtered through 500 µm mesh was divided into four 40 mL aliquots for LC-HRMS analyses. Aliquots were separately centrifuged at 7500 rcf (×g) and 10 °C for 20 min using a Beckman Coulter centrifuge equipped with a JS-7.5 rotor. The supernatants from each of the 4 tubes were removed, placed in 50 mL conical polypropylene tubes and kept frozen until further processing. Prior to the SPE clean-ups, the supernatants were thawed and filtered through 47 mm, 1.2 μm filters (PRAT Dumas, France). Solid-phase extraction (SPE) clean-ups were conducted according to Gutierrez et al. (Gutierrez et al. 2013) with the only modification of the cartridge type and brand. SPE tC18 SEP-PAK® cartridges (500 mg, 3 mL) were obtained from Waters Corporation (Spain).

A volume of 3 mL MeOH was added to each centrifuge tube containing the cell pellets and these tubes were frozen at −24°C for at least 24 h. Subsequently, tubes were placed on ice and cells were sonicated at intervals of 5 × 60 s using an Ultrasonic Homogenizer 4710 series (Cole-Palmer Instruments Co., Chicago, Illinois) set at 45% output control and 100 tune for minimum. Extracts were observed with a light microscope to check that *E. sanguinea* cells were broken. Then, they were further sonicated for 60 s and centrifuged at 7500 rcf (× *g*) and 10 °C for 10 min using the same centrifuge and rotor. Methanolic supernatants were filtered through PTFE 13 mm diameter, 0.22 µm Filter-Lab® (Spain) filters prior to LC-HRMS analyses.

#### Mallard samples processing

Liver, brain, and digestive contents from several mallards pooled in the WRC of Carballedo, as formerly described, were analyzed. Two samples from each of these tissues were obtained from two different mortality events in 2024. The amount of material processed in each case depended on the sample supplied. Whenever possible, 1–2 g of each sample was taken for extraction with 80% MeOH (solvent/sample ratio: 4:1) and homogenized using an IKA Ultraturrax for 3 min. Extracts were then centrifuged (same centrifuge and rotor as before) at 7500 rcf (×g) and 10 °C for 10 min. Sample supernatants were decanted into new tubes and filtered through PTFE 13 mm diameter, 0.22 µm Filter-Lab® (Spain) filters prior to LC-HRMS analyses.

### LC-HRMS equipment and operation

LC-HRMS analyses were conducted using a 1290 Infinity II series UHPLC coupled to a Revenue G6575A Quadrupole Time of Flight (QTOF) spectrometer equipped with a G1958-6527 Jet Stream® ion source (Agilent Technologies, CA, USA). Acquisition was controlled by MassHunter Work Station Software (version 12.0) with the use of Openlab Software (version 3.6). Data analyses were carried out using Agilent MassHunter Qualitative Analysis Software (version 12.0). Analytes were separated on a Zorbax Eclipse Plus C18 rapid resolution HD, 1.8 µm particle size, 50 mm × 2.1 mm i.d. column. Mobile phases were: 90% water, 10% MECN, 0.1% formic acid (A) and 100% MECN, 0.1% formic acid (B).

Initial gradient conditions were 90% A and 10% B and kept for 2 min. Then euglenophycin was eluted over a 6 min gradient from 10 to 90% mobile phase B. This was followed by 3 min at 90% mobile phase B before returning to initial conditions that were kept for 4 min. Flow rate was set at 0.25 mL/min and the column oven was maintained at 40°C. Samples were kept in a thermostatted autosampler at 8 °C. Injection volume was 10 µL. Source conditions were set as follows: source temperature 325 °C, drying gas, N_2_ at 8 l/min flow rate, sheath gas flow rate 11 l/min and temperature 250 °C, capillary voltage 3500 V, nozzle voltage 1000 V, octapole RF 750.

The QTOF instrument was tuned at least monthly and calibrated in positive ion mode before each batch of analyses with the Agilent LC/MS ESI-X Tuning Mix. A 95% MeCN solution containing the reference mass compounds purine and 3H-tetrafluoropropoxylphosphazine was continuously infused during analyses to correct the measured *m/z* values throughout each batch. The instrument was operated in full scan positive mode over a mass to charge ratio (*m/z*) of 100–1000 at a scan rate of 2.5 spectra s^−1^. For qualitative data analyses the Qualitative Software Compound Screening tool was chosen and the “Find by Formula” algorithm was employed. This algorithm takes a target compound’s formula, calculates its theoretical mass and isotopes, and then searches the data file for corresponding extracted ion chromatograms (EICs). The euglenophycin C_20_H_35_NO formula was set as target and the allowed ion species selected were [M+H]^+^, [M+NH_4_]^+^, [M+Na]^+^, and neutral loss of H_2_O. The automated process assigns a probability match score to the identified compounds based on how well they match the expected formula and isotopes.

### Organic pollutants

Persistent Organic Pollutants (POPs), specifically polycyclic aromatic hydrocarbons (PAHs), polychlorinated biphenyls (PCBs), organochlorine pesticides (OCPs), and polybrominated diphenyl ethers (PBDEs), were quantified in both water samples and *Lemna minor* tissues.

Water samples (2 L) were extracted using Oasis HLB solid-phase extraction (SPE) cartridges (500 mg) following the addition of internal standards. Elution was performed with 10 mL of hexane:dichloromethane (1:1, v/v). The resulting extract was concentrated to 1 mL prior to instrumental analysis.

After separation and filtration, 5 g of *L. minor* tissue was extracted using an Accelerated Solvent Extraction (ASE) system using hexane:acetone (1:1, v/v) as the solvent, following the addition of internal standards. The extract was cleaned using deactivated alumina (6% water, 6 g) with hexane as eluent. A portion of the cleaned extract was directly analyzed—for PAHs and PBDEs—while another was fractionated on a silica column (1.6 g, deactivated with 1% water) using 5 mL of isooctane followed by 10 mL of isooctane:diethyl ether (9:1, v/v). Instrumental analysis was performed as follows: PAHs and PBDEs were quantified by gas chromatography coupled to mass spectrometry (GC-MS), and PCBs and OCPs were determined by gas chromatography with electron capture detection (GC-ECD) following the analytical method described in Viñas et al. (2018). Laboratory analyses for PAHs,

PCBs, OCPs, and PBDEs were conducted under strict internal and external quality control procedures. These included the use of reference materials and participation in intercalibration exercises, ensuring the accuracy and reliability of the results.

## Results

### Environmental conditions and biological communities in A Congorza

In recent years a slight increase in the air temperature has been registered in the study area, with +0.5 °C in the maximum and average annual temperatures during 2021–2024 (25.53 °C and 15.81 °C) compared to 2017–2020 (25.05 °C and 15.30 °C). This increase is more pronounced in the minimum temperatures with up to +0.8 °C (8.72 °C vs 7.93 °C). Such variations reflect higher temperatures in the coolest part of the year, and fade out if data for the hot season (May–Sep) are selected for the comparison. In contrast, maximum pluviosity has only marginally increased since 2017, but the period of May–Sep registered maximum values 50% higher in 2021–2024 relative to 2017–2020.

Water surface temperature during four months in spring and summer 2025 (Jun–Sep) oscillated between a minimum of 15.8 °C (Sep 23) to 27.3 °C (July 8) with an average temperature and pH of 20.22 ± 2.19 °C and 6.34 ± 0.31 (Servizo de Patrimonio Natural de Pontevedra, Xunta de Galicia). Maximum average temperatures were recorded in July–August (21.11 and 21.23 °C, respectively).

Parallel samplings between May 29 and Sep 8 (ROAGA-LMAG, Xunta de Galicia) for diverse water quality parameters in surface waters were also available (Table 1). These data evidenced the poor conditions of the pond, with high values for the biological oxygen demand (BOD, range 17–130 mg L^−1^), chemical oxygen demand (COD: 56–535 mg L^−1^), total suspended solids (TSS: 13–320 mg L^−1^), and suboxic conditions (DO: 1.5–5.2 mg L^−1^). Regarding nutrients, NO_3_^−^, NH_4_^+^, and NO_2_^−^ were below quantitation limits, whereas total nitrogen and phosphorus exhibited high values (TN: 1.5–18 mg L^−1^; TP: 0.2–1.7 mg L^−1^). In contrast, one sample taken in Aug 2025 near the bottom in the middle of the pond showed high values for NH_4_^+^ (1.53 mg L^−1^) and H_2_PO_4_^−^ (0.29 mg L^−1^), with NO_3_^−^ and NO_2_^−^ values below quantitation limits of ROAGA-LMAG (in this case TN and TP were not determined).

**Fig. 2:**
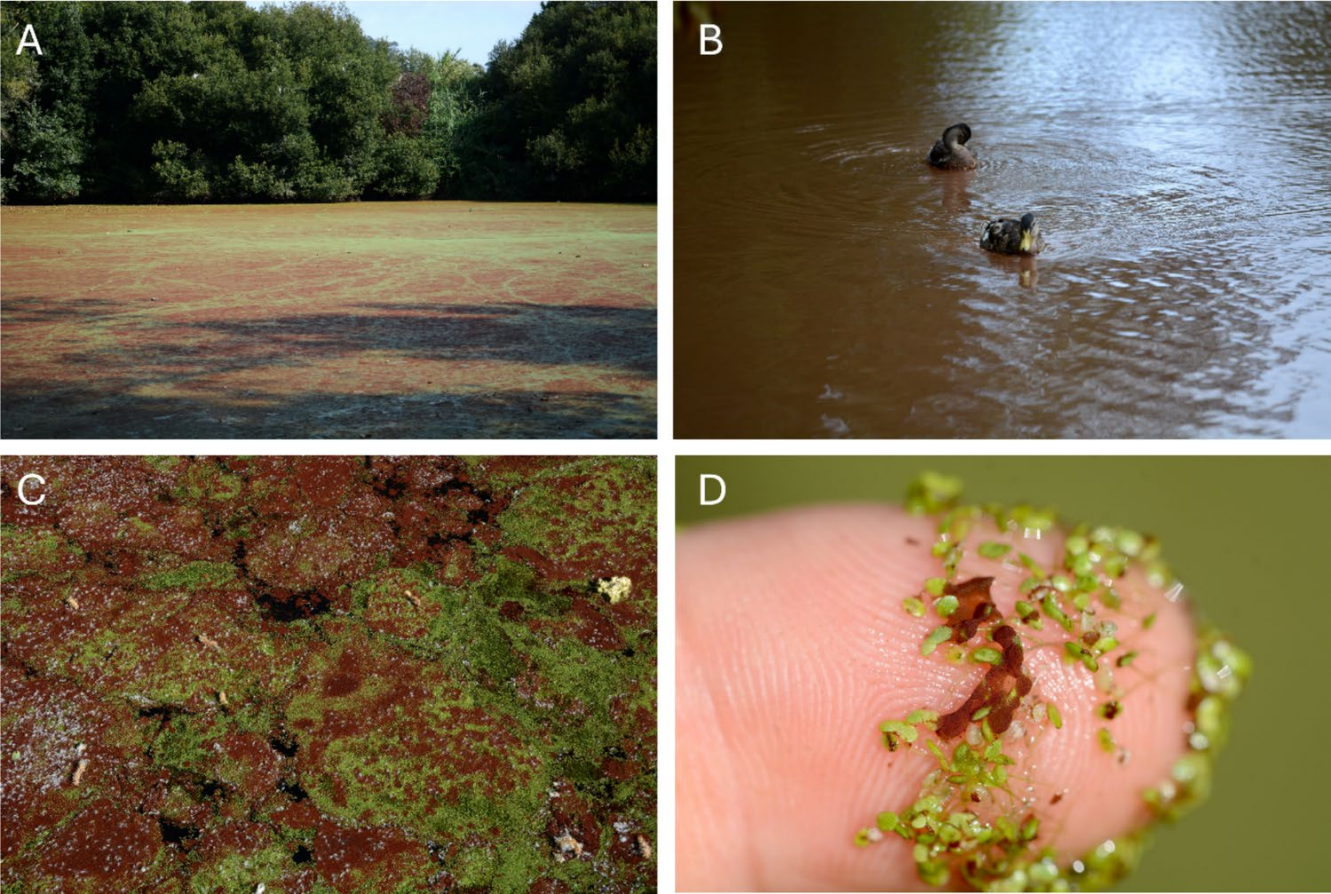
Current view of the pond of A Congorza during summer showing **A** the covering by *Lemna minor* and the red scum, **B** mallards (*Anas platyrhynchos*) in the brownish turbid waters visible when duckweed and algae are displaced by local wind.(Source: A. Fernández) **C** and **D** Detail on *Lemna minor *from A Congorza

**Fig. 3:**
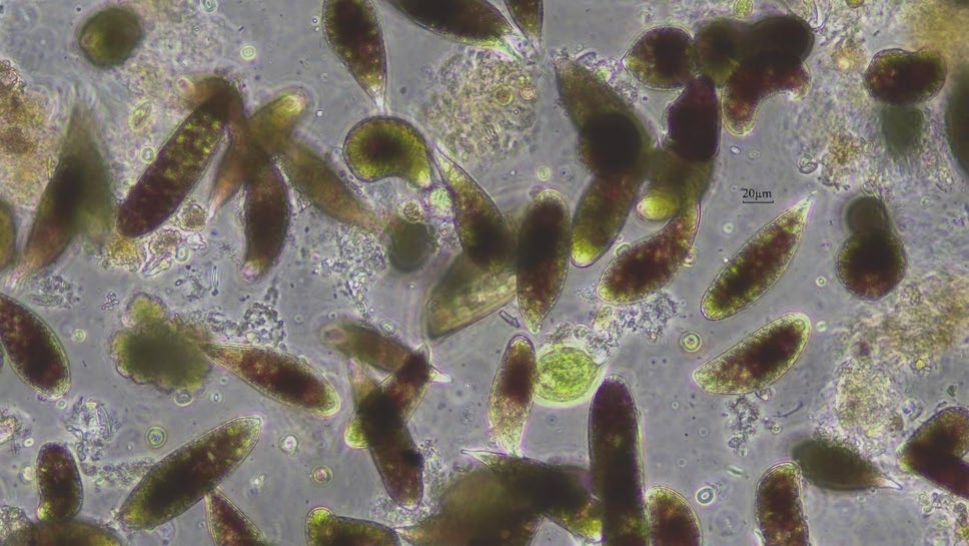
Light micrograph at 200× magnification showing the cells of *Euglena sanguinea* responsible for the reddish hue on the surface of A Congorza

The whole surface of A Congorza was covered by a layer of *L. minor* with variable thickness (Fig. 2). In addition, swarms of a small gastropod (tentatively identified as the invasive species *Physella acuta*; B. Almón (IEO-CSIC) pers. comm.) were also noticed in summer 2025. Green and red scum, stuck to *L. minor*, were harvested from the surface of the pond in summers 2021, 2024, and 2025, containing palmeloid and flagellate stages of an euglenophyte identified by light microscopy as *E. sanguinea* (Fig. 3; video footage in supplementary information: online resource Video S1). Size of both stages, palmeloid and flagellated, ranged from 36 ± 9.1 μm (n = 23) and 120.5 ± 29.2 μm length and from 54.3 ± 3.9 μm width (n = 44), respectively.

Qualitative description of the planktonic community in the surface of the pond during summer 2021, 2024, and 2025, based on light microscopy, showed the presence of heterotrophic taxa with ciliates from the genera *Coleps*, *Euplotes*, *Paramecium*, and *Spirostomum*. Bacterial aggregates were also common. Regarding photosynthetic taxa, cyanobacteria (e.g., *Aphanocapsa*,* Merismopedia*,* Oscillatoria*, and* Planktolyngbya*) and diverse protists including chlorophytes (*Pandorina*), euglenophytes (*Euglena*, *Lepocinclis*, *Menoidium*, *Phacus*, and *Trachelomonas*), and diatoms (*Achnantes*, *Achnantidinium*, *Gogorevia*, and *Lemnicola* (epiphytic on *L. minor*)) were represented.

### Molecular characterization of *Euglena sanguinea*

**Fig. 4:**
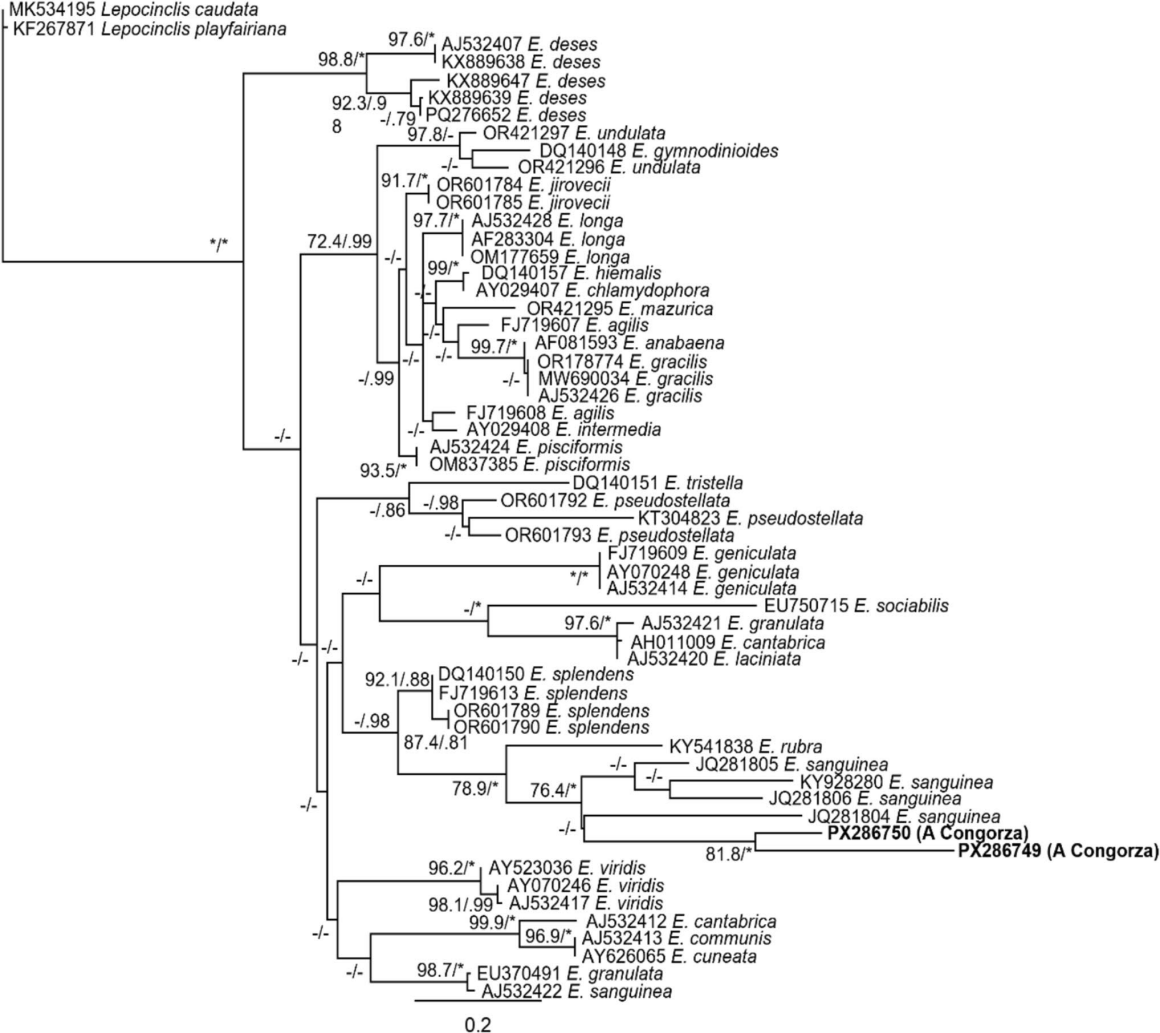
Phylogenetic tree obtained by BI model based on SSU rDNA sequences, showing the relationships between *Euglena sanguinea* cells isolated from the pond of A Congorza (in bold) and other euglenophytes. Numbers on branches are bootstrap percentages (n = 1000) and posterior probabilities after ML and BI analyses, respectively. Asterisks denote maximum statistical support and values lower than 0.70/70% are indicated by hyphens

Phylogenetic analyses of SSU rDNA (Fig. 4) placed the sequence obtained from A Congorza within a clade of *E. sanguinea* containing four sequences with isolates originating from Argentina, UK, and USA (Karnkowska-Ishikawa et al. 2013; Kulczycka et al. 2018). Sequences of *E. sanguinea* were more closely related to *E. rubra* and *E. splendens* (0.301 and 0.313 average base substitutions per site, respectively), branching in a sister clade with other sequences from *E. laciniata*, *E. sociabilis*, and *E. geniculata*. Divergence between *E. sanguinea* sequences (0.27) was slightly lower than those of its closest relatives.

### HPLC pigment analysis of *E. sanguinea*

**Fig. 5:**
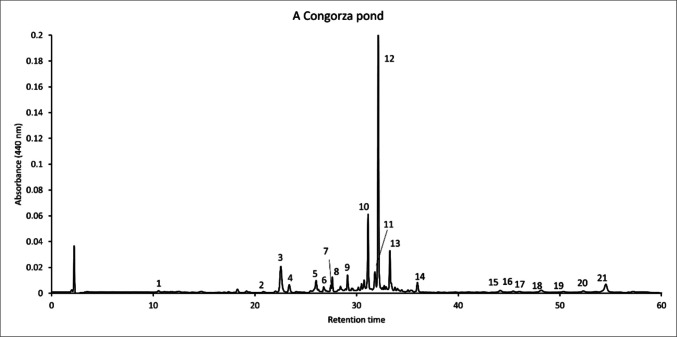
HPLC pigment analysis of a selected representative sample from A Congorza pond surface water (August 2025)

The chromatograms of pigment extracts from the lagoon waters appear dominated by xanthophylls that include at least one ketone group conjugated with the central chain double bond system characteristic of carotenoids (Fig. 5 and Table 3), as demonstrated by their rounded online spectra lacking fine structure.

**Table 3:** Identification of major pigments detected using HPLC analysis

Peak	Retention time (min.)	Vis maxima (nm)	Identification (t: tentative)
1	10.5	430, 581, 663	Chlorophyllide *a*
2	20.8	416, 440, 470	Violaxanthin
3	22.5	483	Asthaxanthin
4	23.4	446, 476	Diadinoxanthin
5	26.0	474	Adonirrubin (t)
6	26.8	454, 481	Diatoxanthin
7	27.5	453, 478	Zeaxanthin
8	27.6	446, 475	Lutein
9	29.1	474	Adonirrubin monoester (t)
10	31.1	465	Adonixanthin monoester (t)
11	31.8	462, 599, 648	Chlorophyll *b*
12	32.1	465	Adonixanthin monoester
13	33.3	432, 617, 662	Chlorophyll *a*
14	36.0	454, 478	β,β-Carotene
15	44.1	483	Asthaxanthin diester (t)
16	45.4	463	Adonixanthin diester (t)
17	46.0	483	Asthaxanthin diester (t)
18	48.7	483	Asthaxanthin diester (t)
19	50.3	483	Asthaxanthin diester (t)
20	52.3	483	Asthaxanthin diester (t)
21	54.5	465	Adonixanthin diester (t)

The corresponding peaks were identified respectively as astaxanthin (for which an authentic standard was available), adonirubin and adonixanthin, and their mono- and diesters that retain the spectral properties of the corresponding free xanthophylls. The presence of chlorophylls *a* and *b*, also abundant, contributes to completing the characteristic marker pigment profile of *E. sanguinea*. Other carotenoids detected include the photoprotective diadinoxanthin, diatoxanthin, violaxanthin, and zeaxanthin. Finally, other trace carotenoids that could not be identified point to the presence in the samples of other planktonic organisms different from *E. sanguinea*. Reddish and greenish samples showed very similar qualitative chromatographic profiles.

### Clinical signs in mallards

**Fig. 6:**
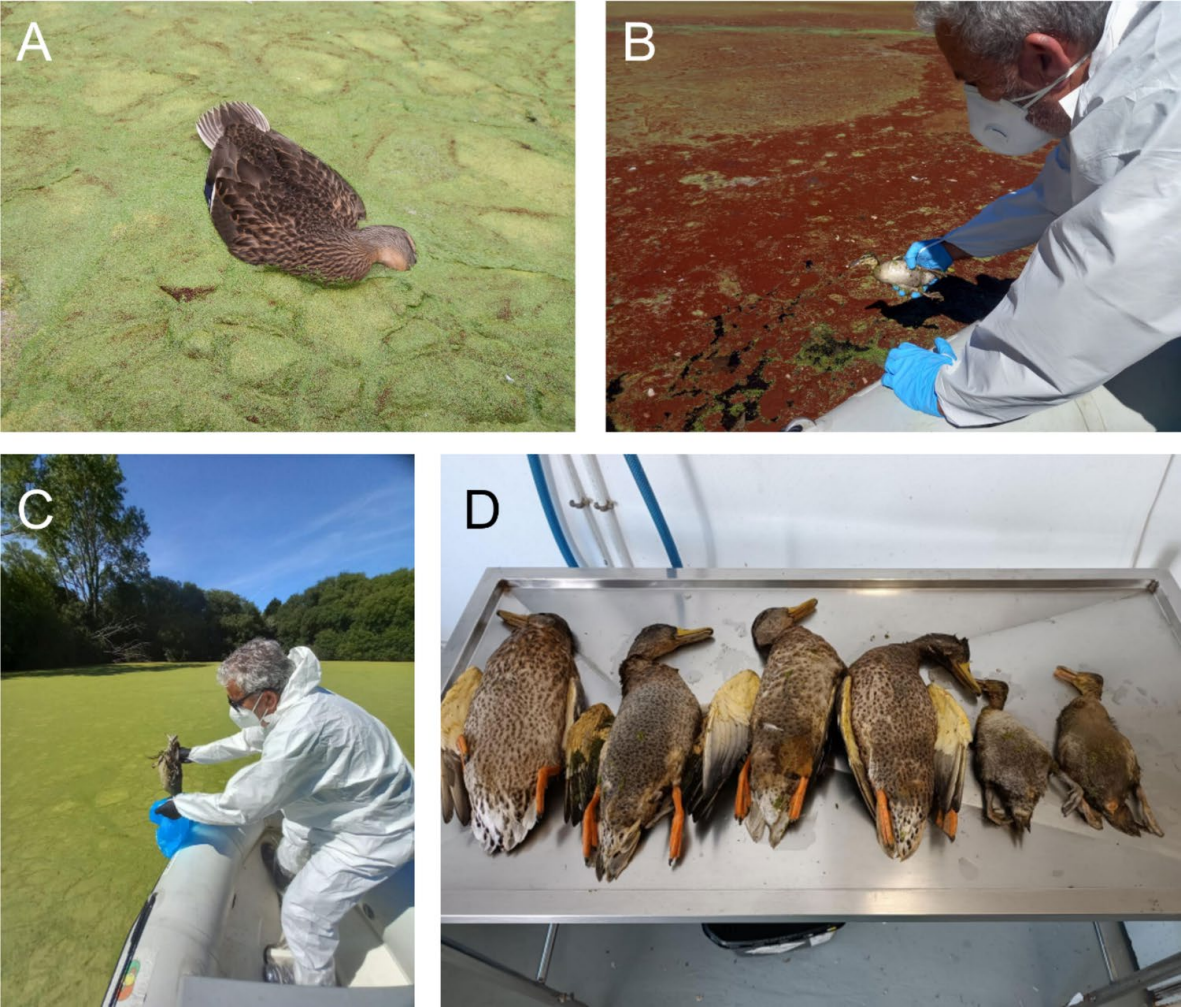
Dead specimens of *Anas platyrhynchos*, **A**–**C** collected in May 2025 from the pond, **D** collected in August 2024 for necropsy in the facilities of WRC Carballedo. Source: Xunta de Galicia

In 2024 and 2025 between 9 and 11 dead mallards were collected by WRC of Carballedo (Table 2; Fig. 6). The number of dead specimens in 2024, before the onset of the surveillance protocol by Xunta de Galicia, could be larger as indicated by local observers (involving males, females, and ducklings; A. Fernández Cordeiro *pers. comm*.), and these carcasses could have sunk in the pond. Between Nov 2024 and May 2025, regular visits by staff of the Natural Heritage Service followed the evolution of waterbirds in A Congorza, dominated by mallards, little egrets, and gray herons (maximum abundance of each around 25 individuals). First sightings of ducklings dated from the beginning of April, prior to the record of a dead female mallard in the centre of the pond on May 24. Within that period, the surface of the pond was covered by a green mat of *L. minor*, partially dispersed by the wind as mentioned before, with no traces of red hues.

The affected mallards typically became motionless in the pond. Their necks progressively lost muscle tone, causing their heads to droop forward as if they were falling asleep. Individuals that remained in the water eventually ended up with their beaks submerged, leading to drowning (A. Fernández Cordeiro, pers. comm.). Rescued mallards by the WRC of Carballedo typically showed normal body temperature, weakness, prostration, and flaccid paralysis of the neck and extremities, compatible with a paralytic syndrome caused by botulinic toxins. Individuals admitted to the WRC facilities first received intravenous and oral fluid therapy with Ringer’s lactate and vitamin supplements. As an example, a duckling rescued in August 2024 exhibited steady clinical improvement. By day 2 of treatment, it had regained head mobility and responded appropriately to external stimuli. By day 4, it was able to stand on its tarsi, and by day 5 it managed intermittent standing and was offered food. On day 8, it was feeding independently, which allowed discontinuation of fluid therapy and oral supplements. By day 11, the bird was transferred to a facility for muscle and weight conditioning, and it was successfully released on day 24. Other individuals with severe clinical signs died after some hours or had to be euthanized (Table 2).

### BoNT, bird diseases, and cyanotoxin analyses

BoNT analyses were performed on tissue samples obtained from dead mallards. The output of BoNT analyses by ELISA assay in 2024 was reported as positive by LABOKLIN (Spain) in maggots and larvae from mallard tissues for BoNT C/D. In 2025, BoNT C in larvae samples from mallards was proved positive by Laboratorio Central de Veterinaria de Algete (Spain). Other bird diseases (West Nile fever (WNF), Newcastle disease, and avian influenza) rendered negative results in the analyses performed by this laboratory.

Finally, LC-MS/MS analyses of cyanotoxins in water samples from summer 2024 showed results under the quantitation limit (0.20 µg L^−1^) for the tested compounds (anatoxin A, cylindrospermopsin, homoanatoxin A, microcystins, and nodularin).

### Euglenophycin analyses

**Fig. 7:**
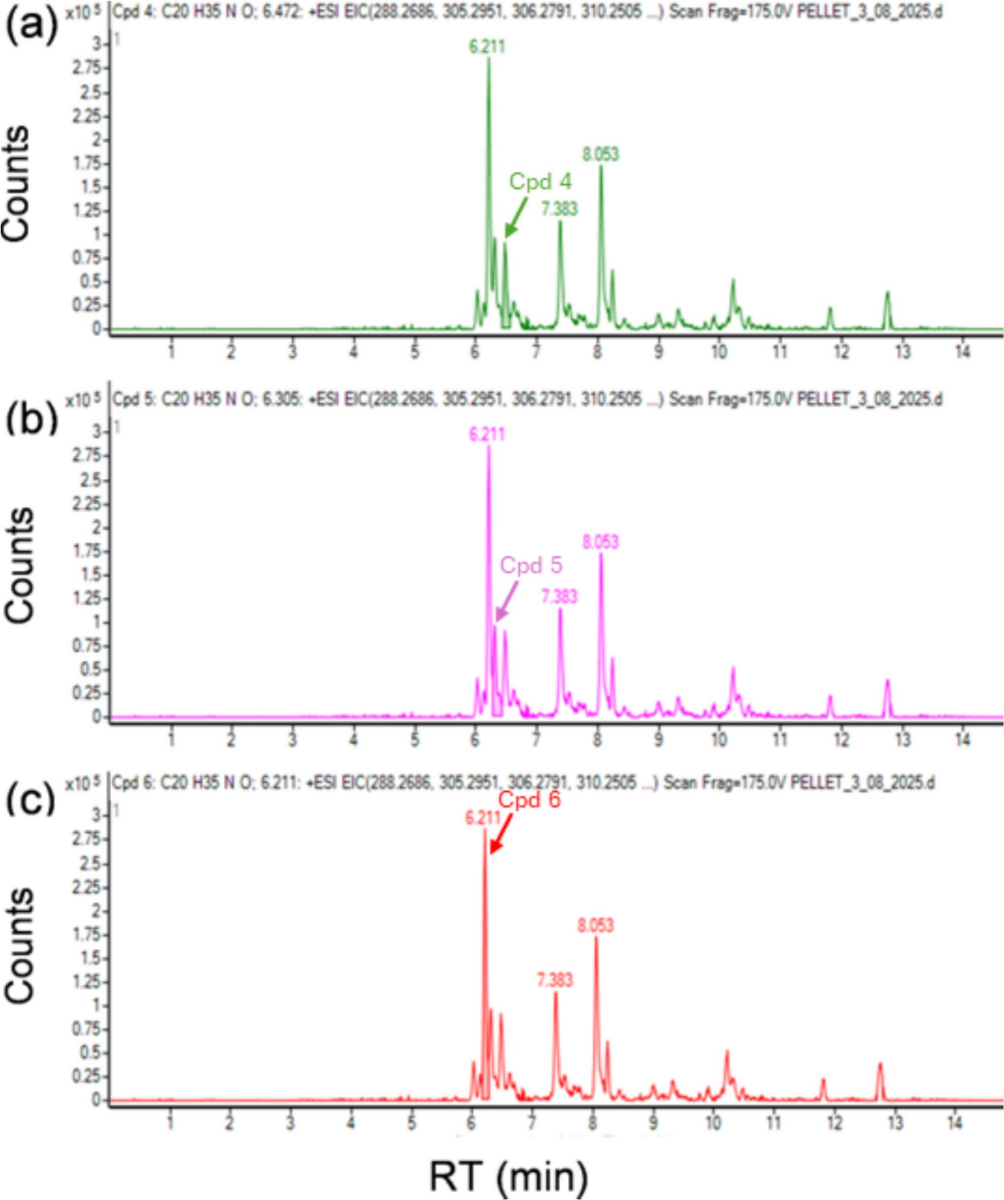
Extracted ion chromatograms of (**a**) compound 4, (**b**) compound 5, and (**c**) compound 6 in sample cells pellet 3 obtained using the FBF algorithm. RT denotes retention time on the X-axis in all three panels

**Fig. 8:**
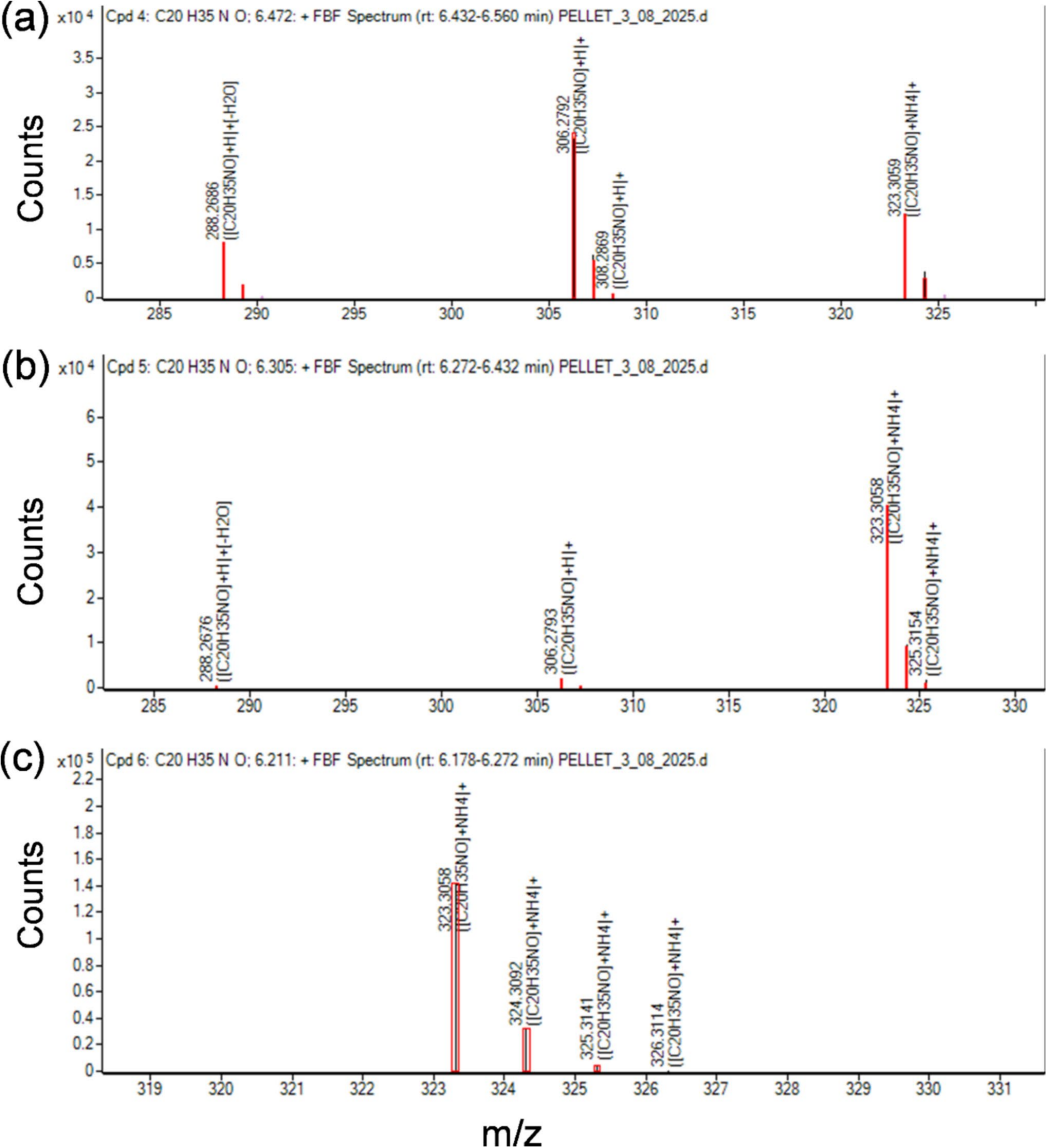
FBF MS spectra of (**a**) compound 4, (**b**) compound 5, and (**c**) compound 6 in sample cells pellet 3

When applying the “Find by Formula” algorithm of the Qualitative Software Compound Screening tool to the cell pellets chromatograms, several compounds were identified as putative euglenophycin. Table 4 summarizes the three compounds in each sample that obtained scores higher than 90% (Figs. 7 and 8). In the four water supernatant samples, none of the peaks corresponding to retention times (RTs) indicated in Table 4 was identified. The Find by Formula Algorithm (FBF) identified other peaks at different RTs (9.0, 9.1, and 10.5 min) but always with probability match scores lower than 88% and they were not identified in all four samples.

**Table 4:** Putative euglenophycine peaks reported per formula, using the Find by Formula Algoritm in MassHunter Qualitative Analysis Software

Sample name	Compound number	Formula	RT	Area	Experimental *m/z*	Theoretical *m/z*	Ions	Score (Tgt)	Diff (Tgt, ppm)
Cells pellet 1	4	[C_20_H_35_NO+H]⁺	6.49	378,787	306.2793	306.2791	9	96.56	−0.01
	5	[C_20_H_35_NO+NH_4_]⁺	6.29	946,213	323.3058	323.3057	7	98.89	0.70
	6	[C_20_H_35_NO+NH_4_]⁺	6.20	207,120	323.3058	323.3057	7	99.83	0.38
Cells pellet 2	4	[C_20_H_35_NO+H]⁺	6.48	190,318	306.2791	306.2791	6	93.14	−0.04
	5	[C_20_H_35_NO+NH_4_]⁺	6.30	506,951	323.3058	323.3057	4	98.90	0.93
	6	[C_20_H_35_NO+NH_4_]⁺	6.21	961,572	323.3059	323.3057	6	99.33	0.71
Cells pellet 3	4	[C_20_H_35_NO+H]⁺	6.47	332,503	306.2792	306.2791	7	95.67	0.40
	5	[C_20_H_35_NO+NH_4_]⁺	6.31	401,731	323.3058	323.3057	6	97.43	0.82
	6	[C_20_H_35_NO+NH_4_]⁺	6.21	881,561	323.3059	323.3057	4	99.13	0.61
Cells pellet 4	4	[C_20_H_35_NO+H]⁺	6.47	262,557	306.2795	306.2791	8	99.22	1.03
	5	[C_20_H_35_NO+NH_4_]⁺	6.30	232,570	323.3061	323.3057	3	97.99	1.45
	6	[C_20_H_35_NO+NH_4_]⁺	6.21	741,910	323.3062	323.3057	4	98.00	1.83

With regard to the mallard tissue samples, none of the peaks corresponding to RTs indicated in Table 4 was identified in brain and liver samples. The software identified a peak at 6.48 min in one of the digestive content samples, but with a probability match score of 76.73% and a Diff of −3.083. In the same sample two additional peaks were identified as putative euglenophycin, one at a RT: 8.12 min with a probability match score of 96.9% and a Diff of 0.93 and another one at a RT: 10.47 min with a probability match score of 97.5% and a Diff of 1.22.

### Organic pollutants

POPs, including PAHs, PCBs, OCPs, and PBDEs, were analyzed in water samples. In all cases, concentrations of the target compounds were below the respective limits of quantification (LOQ), indicating either their absence or presence at levels not quantifiable under the applied analytical conditions (see supplementary Table S2 for a full list of the compounds).

In *Lemna minor* samples, PBDE concentrations were consistently below the limit of quantification (LOQ), while PCBs and OCPs were either below or very close to the LOQ. PAHs showed generally low levels for parent and monoalkylated compounds; however, three groups of alkylated PAHs exhibited comparatively higher concentrations. Specifically, C_2_-naphthalenes (sum of dimethylnaphthalenes) reached 89 ng g⁻^1^ wet weight, C_2_-benzo[a]anthracenes and chrysenes (sum of dimethylated BaA and chrysene) were 26.8 ng g⁻^1^ wet weight, and C_3_-naphthalenes (sum of trimethylnaphthalenes) were 16.9 ng g⁻^1^ wet weight.

## Discussion

Avian botulism has been identified as one of the causes of paralytic or paretic syndrome in waterbirds worldwide (Shin et al. 2010). The first cases of avian botulism (type C) were reported in western USA and Canada, where massive mortalities of North American waterbirds have been recorded (Shayegani et al. 1984), with annual estimates from thousands to millions of individuals (Rocke and Samuel 1999). In that region, mallards have been used as sentinels for epizootiologic studies and management of avian botulism (Evelsizer et al. 2010a, b; Rocke and Brand 1994).

The typical symptoms in sick waterfowl are flaccid paralysis of the muscles followed by respiratory failure and death (Anza et al. 2016). These are similar to the effects produced by certain infectious diseases, nutritional deficiencies, other neurotoxic compounds such as paralytic shellfish toxins (Ben-Gigirey et al. 2021) and several environmental contaminants such as pesticides (reviewed by Sonne et al. 2012). Among the seven types of BoNT, environmental botulism outbreaks have been mostly associated with serotypes C, mosaic C/D, and E (Espelund and Klaveness 2014; Ovelar et al. 2025; Volponi et al. 2024), generally C/D in the case of freshwater waterbirds (Anza et al. 2016).

For instance, in the Iberian Peninsula, the association between the paretic syndrome in waterbirds and BoNT type C/D has been recently suggested in southern Portugal (Mena-Casero et al. 2025), and would explain the mortalities in that region, mostly gulls, but also ducks, in the last decades. Previously, botulism outbreaks have been reported in southern Spain (e.g., Contreras de Vera et al. 1987), but to our knowledge, the largest waterfowl mortalities were found in central Spain, with 13 avian botulism outbreaks (also type C/D) in the wetlands of “La Mancha Húmeda” between 1978 and 2008, causing 20,000 deaths of more than 50 species (Vidal et al. 2013). Among those, mallards and Eurasian coots showed the highest mortality rates.

### A Congorza: a highly (and deadly) polluted aquatic ecosystem

The botulism outbreak in an enclosed pond like A Congorza shares similarities with a former case in summer 2009 in an urban park in Pescara (Italy), with a massive mortality of waterfowl (87%) including 118 mallards and geese due to type C/D botulism (Badagliacca et al. 2018). The typical symptoms described by these authors were flaccid paralysis of skeletal muscles, agonic dyspnea, paralysis concerning muscles of the cervical (Limberneck syndrome), and hind leg districts. Nonetheless, these authors did not mention a proliferation of duckweed in that pond.

The balanced presence of *L. minor* is indeed beneficial for the ecosystem and its characteristics (high nutrient uptake, detoxification, stress tolerance) turn Lemnaceae and this particular species into interesting research models (Basiglini et al. 2018; Devlamynck et al. 2020; Pham et al. 2025; Thingujam et al. 2024; Vulpe et al. 2025). The problem arises when duckweed becomes invasive and proliferates out of control in eutrophic and stagnant ponds like A Congorza. If the eutrophication persists, the efficient absorption of nutrients by *L. minor* leads to its overgrowth. In consequence, its mats contribute to stabilize the water column and the environment is disequilibrated through a cascade of negative effects (block of sunlight and photosynthesis, reduction of oxygen exchange with the atmosphere, consumption of oxygen by duckweed mats when sunlight is not available, further reduction of dissolved oxygen due to the decomposition of organic matter from dead plants and organisms, etc.; Acosta et al. 2021; Pasos-Panqueva et al. 2024). These are the causes behind the onset of anaerobiosis favourable for the spread of *C. botulinum*.

Duckweed is also known to bioaccumulate and degrade PAHs when present at low concentrations in water, and its potential use as a bioremediator has been widely studied. However, when PAH levels in water exceed certain thresholds, these compounds can exert toxic effects, inhibiting plant growth and reducing photosynthetic pigment content (Ekperusi et al. 2020; Kösesakal et al. 2015). In this study, a total of 35 individual PAHs (parent and methylated) and 12 groups of methylated PAHs were analyzed.

Overall, concentrations were low or very low, often close to the limits of quantification. Only three groups of alkylated PAHs exhibited comparatively higher levels: C_2_-naphthalenes (sum of dimethylnaphthalenes, 89 ng g⁻^1^ w.w.), C_2_-benzo[a]anthracenes and chrysenes (sum of dimethylated BaA and chrysene, 26.8 ng g⁻^1^ w.w.), and C_3_-naphthalenes (sum of trimethylnaphthalenes, 16.9 ng g⁻^1^ w.w.).

The predominance of di- and trimethylated compounds, combined with the scarce presence of parent and monomethylated PAHs, suggests a potential petrogenic source of contamination. Crude oils typically contain higher proportions of methylated PAHs compared to parent compounds, whereas pyrogenic sources (e.g., petroleum combustion, forest fires) tend to degrade methylated species, leaving parent PAHs as dominant. An alternative explanation could involve differential degradation rates, with parent and monomethylated compounds being more susceptible to breakdown than their methylated counterparts. Additionally, the greater solubility and volatility of compounds such as naphthalene compared to their methylated homologues may partly account for the observed pattern. Given that PAH concentrations in water were consistently low and only a few groups of methylated PAHs were detected in *L. minor*, the findings may indicate the occurrence of a minor hydrocarbon input to the area. However, the levels observed do not appear sufficient to account for the mallard mortalities recorded in the pond.

In A Congorza, a group of bird watchers (Grupo Anelamento Anduriña) edited a book on its natural values (Fernández Cordeiro et al. 2006) and their observations allowed them to detail the timeline of its degradation (Fernández Cordeiro A., pers. comm.). First, an episode of great proliferation of duckweed in the years 2003–2004 covered practically the entire pond (paralleling the introduction of domestic ducks and geese). In that period and until 2006 the only fish species in the pond (*Anguilla anguilla*) declined and finally disappeared. The duckweed faded away between 2007 and 2018, but in the summer of that year it proliferated at the bottom of the pond occupying a third of it. So far, the water did not present any abnormal color or turbidity. From the summer of 2019 onwards, duckweed has covered almost the entire surface of the pond, a pattern that has recurred annually. In November and December 2020, the water turned brown and by spring 2021 it had already become dark brown, a condition that persists to the present day. From the summer of 2021, red proliferations began to appear on the duckweed, and by August they covered nearly the entire pond. Several years later, in 2024, the first episodes of duck mortality were recorded.

These mortality events, occurring during the summer–autumn of 2024 and the spring–summer of 2025, coincided with environmental conditions—dry weather and high temperatures—that, together with anaerobic conditions, are conducive to the development of *C. botulinum*. The repeated occurrence of botulism outbreaks can promote the presence of spores in the digestive tract of waterbirds and enhance *C. botulinum* toxigenesis in bird carcasses (known as the “carcass-maggot cycle”). In the studied pond, prior to the implementation of the surveillance protocol by the Xunta de Galicia, mallards that could not be collected immediately after death may have sunk to the bottom, thereby constituting additional sources of *C. botulinum* within this already degraded ecosystem. It should be noted that mallards undergo their moult in A Congorza during the summer, and until their wing plumage is fully restored they are unable to fly, remaining in and feeding within the pond. This circumstance likely makes them even more vulnerable than other waterfowl to the adverse conditions prevailing in such a deteriorated environment. Although avian botulism was confirmed through toxin detection and clinical evidence, the presence of *C. botulinum* in environmental samples was not directly assessed in this study.

### *Euglena sanguinea* blooms and their consequences in A Congorza

To our knowledge, the presence of *E. sanguinea* in Spain was first documented by Álvarez-Cobelas (1984) and it has been then identified as responsible for some reddish blooms, in reservoirs of the Basque Country, Catalonia, Castile and León, and Castile La Mancha (Cambra 1992; Conforti et al. 2005; Estudios y Proyectos Línea 2010; Laza-Martínez et al. 2019). In the studied pond, on top of the deleterious effects driven by duckweed, the mallard mortalities could result from multifactorial causes like feeding on the recurrent potentially toxic proliferations of *E. sanguinea* that coexist with *L. minor* during summer.

In the past, the lack of adequate diagnostic characters led to designate up to 12 species resembling *E. sanguinea*, but a modern redescription reduced these to three: *E. laciniata*, *E. sociabilis*, and *E. splendens* (Karnkowska-Ishikawa et al. 2013). The morphological observations and molecular analyses in the present study using specific primers for the amplification of *E. sanguinea* (Kulczycka et al. 2018) allowed identification of this species as the dominant euglenophyte in A Congorza, responsible for the reddish hue in surface waters. Cell length and width of *E. sanguinea* are the largest among these four species (Karnkowska-Ishikawa et al. 2013). Measurements from individuals in A Congorza were in the upper range (or over) of the average values reported for *E. sanguinea* by these authors (between 80.5–115.1 µm and 22.1–26.9 µm respectively for strains Argentina, Henderson, and SAG 1224–30, the three of them included in our phylogeny with Acc. Nos. JQ281804, JQ281805, and JQ281806, respectively). In contrast, *E. sociabilis*, *E. splendens*, and *E. laciniata* had average cell values of 53.8–89.4 µm in length and 12.2–27.0 µm in width (Karnkowska-Ishikawa et al. 2013). It cannot be discarded that other *Euglena* species were present in the bloom, but two independent PCR amplifications with specific primers (Kulczycka et al. 2018) retrieved sequences for *E. sanguinea*. In addition, repeated efforts with PCR assays using universal primers for nSSU rDNA were always unsuccessful. This could be another indication about the dominance or monospecific nature of the bloom, given that *E. sanguinea* is the only species of its genus for which standard methods of nSSU rDNA amplification have proved unsuccessful due to the unusual structure of its sequence (Kulczycka et al. 2018).

Regarding photosynthetic pigment composition, although the lack of standards for adonirubin and adonixanthin prevented certainty in their identification, the predominance of their esters, together with those of astaxanthin, is strongly supported by their chromatographic behavior and spectral properties and agrees with the previously described occurrence of these compounds in *E. sanguinea* pigment extracts (Grung and Liaaen-Jensen 1993; Laza-Martínez et al. 2019). However, none of these ketonic xanthophylls nor their esters were detected by Deli et al. (2014) in a natural bloom of this species in which the acetylenic diatoxanthin and its esters seem to replace the ketonic carotenoids. Although biological differences could underlie this result, the use of drastic saponification conditions that could have led to astaxanthin alteration (Eugster 1995; Yuan and Chen 1999) may explain the degradation of esters whose spectral properties, far from matching the fine spectral structure of diatoxanthin, resemble those of ketocarotenoids.

The simultaneous detection during blooms of *E. sanguinea* in A Congorza of carotenoids of the epoxidation/deepoxidation cycles of both the red (diadinoxanthin and diatoxanthin) and green (violaxanthin and zeaxanthin) lineages supports previous results (Roach et al. 2015) and points to the coexistence of both cycles in the complex pigment systems of euglenophytes (Laza-Martínez et al. 2019). However, the isolation of the strain and the growth of monoalgal cultures would be necessary to ascertain this fact. In any case, the main photoprotective mechanism of this species has been demonstrated to be based more on the movement of red coloured organelles towards the periphery of the cells (thus decreasing the light energy reaching the photosynthetic apparatus) than on energy dissipation by the xanthophyll cycles (Laza-Martínez et al. 2019) and this explains the fact that the pigment profiles do not essentially differ in samples of the red and green *E. sanguinea* scums in A Congorza.

Toxic blooms of euglenophytes are known to cause problems in freshwater due to shade of vegetation and incoming light in the water column, a phenomenon that affects the entire pond of A Congorza due to the massive growth of *L. minor*. In A Congorza, the surface bloom of *E. sanguinea* competes with *L. minor* for nutrients, contributing to the blocking of sunlight, the reduction of oxygen exchange due to its scum and the consumption of oxygen when light is not available. Dense blooms of *E. sanguinea* also deplete oxygen levels during the decay of their populations (Sultana et al. 2024 and references therein), worsening the ambient conditions that favour the development of *C. botulinum*.

The factors conducive to euglenophyte proliferations include sunlight, water temperature, pH and excessive nutrients, in particular the presence of high nitrogen and phosphorus concentrations, heavy metals, acidic pH, and temperatures over 20 °C (Sultana et al. 2024 and references therein). Most of these conditions prevailed in the studied pond. However, the potential influence of heavy metals, which were not analyzed in this study, could not be assessed. POPs were analyzed and detected at low, non-lethal levels; however, their ecological role in promoting euglenophyte proliferations remains poorly understood. Previous studies suggest that euglenoids may actively interact with these compounds. For instance, euglenoids such as *Trachelomonas* sp. and *Euglena* sp. have been reported to exhibit a high uptake capacity for PCBs, likely associated with the presence of a β−1,3-glucan storage product that plays a key role in the selective sorption of PCB congeners (Fitzgerald and Steuer 2006). This capacity could influence both the bioavailability and ecological dynamics of POPs in aquatic systems.

Previous studies have linked euglenophyte occurrence to organic enrichment and eutrophic conditions. Several species of Euglena, including *E. gracilis* and *E. viridis*, have been reported as characteristic taxa in organically polluted waters, sewage retention basins, and nutrient-rich environments (Palmer 1980). Similarly, photosynthetic euglenophytes are widely regarded as indicators of organic pollution because of their tolerance to elevated nutrient loads and their mixotrophic metabolism (Arguelles and Martinez-Goss 2014).

UHPLC-HRMS analysis revealed several peaks in the cell pellet samples that were consistent with putative euglenophycin. Zimba et al. (2010) purified and isolated several toxic compounds, from *E. sanguinea* batch cultures, that showed diagnostic peaks of 306 (M+H)^+^ and 288 (M+H-H_2_O)^+^ when analyzed by LC-MS/MS. In the present study, both ions were found in the spectra from compounds 4 and 5 (Table 4 and Fig. 8a and b), together with the ammonium adduct, whereas compound 6 (Fig. 8c) showed only the ammonium adduct. The fact that we found more than one putative euglenophycin compound is in agreement with Zimba et al. (2010) findings in their purified extracts, since they reported the presence of several stereo and structural isomers of a single class euglenoid toxic compound with identical mass.

Interestingly, the detection of putative euglenophycin in A Congorza may have broader biogeographical implications. To date, toxic blooms associated with *E. sanguinea* have only been reported in inland waters of the USA, despite the species itself being globally distributed (Patiño et al. 2023). Therefore, if confirmed, our results could represent the first evidence of a potentially toxigenic *E. sanguinea* bloom outside that region. Nonetheless, this interpretation must remain cautious because the identification is based on LC-HRMS only and could not be confirmed with a standard or laboratory clonal cultures. Whether the apparent geographic restriction of these toxic blooms reflects environmental factors, genetic differences among strains, or simply limited monitoring effort remains unknown. Further work combining toxin confirmation and strain characterization will be necessary to clarify the global distribution and ecological relevance of toxigenic euglenophytes.

Euglenophycin is an ichthyotoxic alkaloid with reported herbicidal activity and inhibitory effects on mammalian tissue growth, produced by several strains of *E. sanguinea* (Zimba et al. 2017). However, its toxicological effect on birds is yet to be determined. Accordingly, while we cannot exclude the possibility that *E. sanguinea* blooms represented an additional stressor in this degraded system, there is no direct evidence that euglenophycin contributed to the observed mallard mortalities, for which avian botulism remains the primary confirmed cause.

### Management of the environment in A Congorza

At present, the surveillance protocol by the regional government (Xunta de Galicia) includes a regular monitoring of water parameters between May and October (temperature, pH) and other physico-chemical parameters (oxygen, nutrients, etc. as listed in Table 1) and the removal of floating bird carcasses and the rescue of sick mallards. These must be associated with counteracting methods to stop its gradual degradation with the objective of restoring the ecological equilibrium in the pond and the health of its ecosystem.

The European Water Resilience Strategy entails the protection and restoration of aquatic ecosystems in the EU (European Commission 2025; European Environment Agency 2025). One of its key objectives is restoring and protecting the water cycle as a basis for sustainable water supply. That document specifically mentions that limiting the pollution of aquatic ecosystems by nutrients should be placed at the centre of restoring water quality. These inputs from agriculture, urban settlements, etc. impact human health and cause algal blooms and oxygen-depletion lethal to aquatic ecosystems posing a major challenge with socio-economic annual losses between 75 and 485 billion euros (Van Grinsven et al. 2013).

The water quality parameters determined in the pond matched those typical of organic pollution from wastewater evidencing the disruption of its natural balance. A malfunction of the sanitation network could lie at the heart of such disturbance, worsened by the ongoing degradation of an enclosed ecosystem. This should be urgently examined by the authorities to put into place the necessary corrective measures. The Urban Waste Water Treatment Directive (EU/2024/3019 (2024), a recast of the previous Directive 91/271/EEC (1991) included within the programmes of measures by the EU Water Framework Directive (WFD) 2000/60/EC (2000)), lists the requirements for discharges from urban wastewater treatment plants. There, the concentration values set for BOD, COD, and total suspended solids are fixed at 25, 125, and 35 mg L^−1^, respectively, for agglomerations of 10,000–150,000 population equivalents. Such figures are the case for A Congorza, located in Cangas do Morrazo with 26,714 inhabitants (INE 2024). In the studied pond, during the period between May 29 and Sep 1 in 2025, these values were often surpassed (Table 1) and exhibited several maxima compatible with untreated wastewater suggesting an inefficient functioning in the sanitary network. Regarding total nitrogen (TN) and phosphorus (TP), the values set by that Directive (EU/2024/3019; 10 and 0.7 mg L^−1^) were only overcome on July 9 and some dates in August for TP. Therefore, A Congorza can be described as a severely degraded aquatic system with wastewater-like characteristics. It did not meet the requirements even for urban wastewater in the EU during the studied period, evidencing an alarming condition that explains the harms to aquatic life (absence of fish, onset of euglenophyte and *L. minor* blooms) and waterfowl deaths.

This environmental issue should be taken into serious consideration by the local authorities in order to minimize the pollution suffered by the pond as a requisite to recover the natural equilibrium with further measures. Without managing its water pollution, any efforts in A Congorza will be useless or even counterproductive. For instance, if *L. minor* populations are directly removed the current scenario of eutrophication could trigger toxic cyanobacterial blooms as those affecting other water reservoirs in Galicia (e.g., As Conchas due to the effects of intensive livestock farming; Parliamentary question E-002600/2025 submitted 27.6.2025; europarl.europa.eu/doceo/document/E-10–2025−002600_EN.html).

A local environmental project (Santos-Tenreiro 2024) summarized four management solutions that the authors of this study strongly support: (i) to reduce the excess of nutrients that enter the pond through wastewater; (ii) bubbling of the pond to introduce agitation that will aerate the water and reduce growth of *L. minor*; (iii) restoration of the ecosystem (clean-up of bottom sediments and exotic plants, management of adequate water levels in the pond during the year); and (iv) dissemination and valorisation of the ecosystem including informative panels with the natural values of the ecosystem and recommendations for its safe observation and conservation. Last but not least, removal of carcasses should be carried out to deter the “carcass-maggot cycle” responsible for the spread of *C. botulinum*.

## Conclusions

The environmental objectives of the WFD state that EU countries shall implement the necessary measures to prevent deterioration of the status of all bodies of surface water. Member States must urgently implement all measures to achieve a good ecological status to meet the WFD’s 2027 deadline but only 39.5% of surface waters meet such a goal according to the “Ecological status of surface waters in Europe” published online by the European Environment Agency on Sep 2, 2025. Therefore, it is urgent to establish measures focusing on the restoration of the water quality of A Congorza, which will be essential as a first step towards managing its whole ecosystem, counteracting its degradation and harmful effects to wildlife and domestic animals.

To that aim, once the source of polluted water is identified and removed, other measures could be put into practice: e.g., periodic removal of a fraction of the duckweed biomass as a first approach can be helpful, but always accompanied by additional measures to prevent their overgrowth (e.g., water movement and aeration). Not to mention that given the surface of the pond (5300 m^2^) and the current extent of the *L. minor* coverage, we estimated an approximate biomass of 6 Tm of duckweed in A Congorza (based on a weight of 25 g for a 15 × 15 cm mat of *L. minor*). If none of these measures is taken, given that *L. minor* typically reproduces fast and asexually, the excess of nutrients will continue supporting their invasion in the pond. Its partial removal and control would enhance photosynthesis in the water column, introducing new microalgal competitors for nutrients, activating the release of dissolved oxygen and its atmospheric exchange, gradually restoring the ecological equilibrium of the pond, the aquatic life and waterfowl.

## Supplementary Information

Below is the link to the electronic supplementary material.

ESM1 (MP4 203 MB)

ESM2 (DOCX 13.9 KB)

## Data Availability

All data generated or analyzed during this study are included in this published article and its supplementary information files.
