# Supplementary material for: The perfect storm in a coastal pond: duckweed, botulism, bird mortalities, and recurrent blooms of Euglena sanguinea
Source: Environ Sci Pollut Res Int. 2026 May 8;33(16):7933–53. doi: 10.1007/s11356-026-37791-z (PMC13190810; doi:10.1007/s11356-026-37791-z)
Supplement: Supplementary file 2 — (DOCX 13.9 KB) [file 11356_2026_37791_MOESM2_ESM.docx]

**Supplementary information: Video S1**. Video footage of *Euglena sanguinea* bloom in the pond of A Congorza

**Supplementary Table S1**. Gradient profile employed in HPLC pigment analysis (A: methanol, B: acetonitrile, C: aqueous pyridine solution (0.025 M pyridine, pH adjusted to 5.0 with acetic acid) and D: acetone).

| **Time (min.)** | **% solvent A** | **% solvent B** | **% solvent C** | **% solvent D** |
| --- | --- | --- | --- | --- |
| 0 | 50.0 | 25.0 | 25.0 | 0.0 |
| 22 | 38.0 | 39.0 | 15.0 | 8.0 |
| 28 | 21.4 | 58.3 | 1.3 | 19.0 |
| 45 | 21.4 | 58.3 | 1.3 | 19.0 |
| 55 | 20.0 | 50.0 | 0.0 | 30.0 |
| 59 | 50.0 | 25.0 | 25.0 | 0.0 |
| 60 | 50.0 | 25.0 | 25.0 | 0.0 |

**Supplementary Table S2**. Organic pollutants. Individual compounds measured in the samples, including their retention time, quantification ion and Limit of quantification (LOQ) both in water (w) and tissue (t).

| Group | Compounds | Retention time (min) | Quantification Ion | LoQ |
| --- | --- | --- | --- | --- |
| PAHs | Naphthalene (Naph) | 21,93 | 128 | 0.002 ng/ml (w)  0.60 ng/g (t) |
|  | 2-Methylnaphtalene (2M-Naph) | 24,87 | 142 |  |
|  | 1-Methylnaphthalene (1M-Naph) | 25,26 | 142 |  |
|  | Biphenyl (Biph) | 26,73 | 154 |  |
|  | 2,3-Dimethylnaphthalene (2,3 DM-Naph) | 28,37 | 156 |  |
|  | Acenaphthylene (Acy) | 28,67 | 152 |  |
|  | Acenaphthene (Ace) | 29,42 | 154 |  |
|  | 2,3,6-Trimethylnaphtalene (2,3,6 TM-Naph) | 30,88 | 170 |  |
|  | Fluorene (Flu) | 31,85 | 166 |  |
|  | Dibenzothiophene (DBT) | 35,91 | 184 |  |
|  | Phenanthrene (Phe ) | 36,64 | 178 |  |
|  | Anthracene (Ant ) | 36,97 | 178 |  |
|  | 4-Methyldibenzothiophene (4M-DBT) | 38,25 | 198 |  |
|  | 2-Methylphenanthrene (2M-Phe) | 39,45 | 192 |  |
|  | 2,8-Dimethyldibenzothiophene (2,8-DBT) | 39,45 | 212 |  |
|  | 1,6-Dimethylphenanthrene (1,6 DM-Phe) | 41,31 | 206 |  |
|  | Fluoranthene (Fla) | 43,36 | 202 |  |
|  | 2,4,7-Trimethyldibenzothiophene (2,4,7-TM-DBT) | 43,59 | 226 |  |
|  | Pyrene (Pyr) | 44,70 | 202 |  |
|  | 1,2,8-Trimethylphenanthrene (1,2,8 TM-Phe) | 46,92 | 220 |  |
|  | 1-Methylpyrene (1M-Pyr) | 48,07 | 216 |  |
|  | Benzo(c)phenanthrene (B(c)-Phe) | 50,54 | 228 |  |
|  | Benz(a)anthracene (B(a)-Ant) | 51,95 | 228 |  |
|  | Triphenylene (Triph) | 52,09 | 228 |  |
|  | Chrysene (Chry) | 52,20 | 228 |  |
|  | 2-Methylchrysene (2M-Chry) | 54,70 | 242 |  |
|  | 7,12-DimethylB(a)anth (7,12 DMB(a)-Ant) | 58,10 | 256 |  |
|  | Benzo(b)fluoranthene (B(b)F) | 58,28 | 252 |  |
|  | Benzo(k)fluoranthene (B(k)F ) | 58,39 | 252 |  |
|  | Benzo(a)pyrene (B(a)-Pyr ) | 59,79 | 252 |  |
|  | Benzo(e)pyrene (B(e)-Pyr ) | 60,14 | 252 |  |
|  | Perylene (Per ) | 60,59 | 278 |  |
|  | Dibenzo(a,h)anthracene (Db(ah)-Ant ) | 67,10 | 276 |  |
|  | Indeno (1,2,3-c,d) pyrene (IP ) | 67,26 | 276 |  |
|  | Benzo (g,h,i) perylene (B(ghi)-Per ) | 69,33 | 69 |  |
| PBDEs | BDE28 | 27,10 | 79, 81 | 0.0002 ng/ml (w)  0.04 ng/g (t) |
|  | BDE47 | 36,55 | 79, 81 |  |
|  | BDE66 | 37,47 | 79, 81 |  |
|  | BDE100 | 41,18 | 79, 81 |  |
|  | BDE99 | 42,31 | 79, 81 |  |
|  | BDE85 | 44,38 | 79, 81 |  |
|  | BDE154 | 45,57 | 79, 81 |  |
|  | BDE153 | 47,41 | 79, 81 |  |
|  | BDE183 | 54,76 | 79, 81 |  |
| PCBs | PCB-31 | 16,32 |  | 0.0003 ng/ml (w)  0.06 ng/g (t) |
|  | PCB-28 | 16,41 |  |  |
|  | PCB-52 | 18,06 |  |  |
|  | PCB-101 | 24,36 |  |  |
|  | PCB-149 | 31,36 |  |  |
|  | PCB-118 | 31,59 |  |  |
|  | PCB-153 | 35,00 |  |  |
|  | PCB-105 | 35,60 |  |  |
|  | PCB-138 | 39,89 |  |  |
|  | PCB-187 | 43,43 |  |  |
|  | PCB-156 | 50,50 |  |  |
|  | PCB-180 | 52,91 |  |  |
|  | PCB-170 | 55,97 |  |  |
|  | PCB-194 | 61,34 |  |  |
| OCPs | HCB | 13,68 |  |  |
|  | Aldrín | 19,36 |  |  |
|  | αHCH | 13,36 |  |  |
|  | yHCH | 14,34 |  |  |
|  | Trans Clordano | 23,90 |  |  |
|  | Cis Clordano | 25,31 |  |  |
|  | p,p' DDE | 27,26 |  |  |
|  | p,p' DDD | 32,65 |  |  |
|  | o,p' DDT | 33,28 |  |  |
|  | p,p'DDT | 39,02 |  |  |
